# Hyoscyamine in Delirium Tremens

**Published:** 1887-01

**Authors:** 


HYOSCYAMINE IN DELIRIUM TREMENS.—Dr. F. Tipton, of Selma, Ala., has had most satisfactory results with Merck's hyoscyamine, in doses of 1-20 grain hypodermically administered, in the treatment of all the distressing symptoms of delirium tremens. He writes: "It has never yet failed me, nor have I experienced any bad results from this drug in these doses. I treat all cases of delirium tremens with this drug as soon as chloral and the bromides fail, and they all get well rapidly. I use the following formula, which I obtained from Dr. P. Brice, of the Alabama State Insane Asylum:

| | | |
|---|---|---|
| ℞ | Hyoscyamin, Merck's, - - - | gr. j |
| | Alcoholis, - - - - - | ʒ j |
| | Aquæ, - - - - - | ʒ j |

M. Sig.—Inject from five to ten minims for one injection."

